# Microbiota in adult perianal abscess revealed by metagenomic next-generation sequencing

**DOI:** 10.1128/spectrum.03474-23

**Published:** 2024-02-22

**Authors:** Jian-Chen Hong, Jian-Sheng Chen, Zai-Jie Jiang, Zhi-Chuan Chen, Ning Ruan, Xiang-Ping Yao

**Affiliations:** 1 Department of Gastrointestinal Surgery, The First Affiliated Hospital, Fujian Medical University, Fuzhou, China; 2 Department of Anorectal Surgery, National Regional Medical Center, Binhai Campus of the First Affiliated Hospital, Fujian Medical University, Fuzhou, China; 3 Department of Neurology, Institute of Neurology of First Affiliated Hospital, Institute of Neuroscience, Fujian Key Laboratory of Molecular Neurology, Fujian Medical University, Fuzhou, China; 4 Department of Neurology, National Regional Medical Center, Binhai Campus of the First Affiliated Hospital, Fujian Medical University, Fuzhou, China; Department of Pathology, City of Hope, Duarte, California, USA

**Keywords:** perianal abscess, metagenomic next-generation sequencing, microbiota, *Bilophila wadsworthia*, *Escherichia coli*

## Abstract

The microbiota of perianal abscesses is scarcely investigated. Identifying causative bacteria is essential to develop antibiotic therapy. However, culture-based methods and molecular diagnostics through 16S PCR technology are often hampered by the polymicrobial nature of perianal abscesses. We sought to characterize the microbiota composition of perianal abscesses via metagenomic next-generation sequencing (mNGS). Fourteen patients suffering from perianal abscesses between March 2023 and August 2023 underwent retrospective assessment. Information from medical records was used, including clinical information, laboratory data, and culture and mNGS results. Forty bacterial taxa were identified from perianal abscesses through mNGS, with *Bilophila wadsworthia* (71.4%), *Bacteroides fragilis* (57.1%), and *Escherichia coli* (50.0%) representing the most prevalent species. mNGS identified an increased number of bacterial taxa, with an average of 6.1 compared to a traditional culture-based method which only detected an average of 1.1 in culture-positive perianal abscess patients, predominantly *E. coli* (75.0%), revealing the polymicrobial nature of perianal abscesses. Our study demonstrates that a more diverse bacterial profile is detected by mNGS in perianal abscesses, and that *Bilophila wadsworthia* is the most prevalent microorganism, potentially serving as a potential biomarker for perianal abscess.

**IMPORTANCE:**

Accurately, identifying the bacteria causing perianal abscesses is crucial for effective antibiotic therapy. However, traditional culture-based methods and 16S PCR technology often struggle with the polymicrobial nature of these abscesses. This study employed metagenomic next-generation sequencing (mNGS) to comprehensively analyze the microbiota composition. Results revealed 40 bacterial taxa, with *Bilophila wadsworthia* (71.4%), *Bacteroides fragilis* (57.1%), and *Escherichia coli* (50.0%) being the most prevalent species. Compared to the culture-based approach, mNGS detected a significantly higher number of bacterial taxa (average 6.1 vs 1.1), highlighting the complex nature of perianal abscesses. Notably, *Bilophila wadsworthia* emerged as a potential biomarker for these abscesses. This research emphasizes the importance of mNGS in understanding perianal abscesses and suggests its potential for improving diagnostic accuracy and guiding targeted antibiotic therapy in the future.

## INTRODUCTION

Perianal abscesses represent an acute suppurative infection of the soft tissue surrounding the rectum and anus. Perianal abscesses are caused by infected anal glands at the base of the anal crypts and may result in systemic infection and life-threatening sepsis (1). Perianal abscesses are twice as common in men as in women, occurring at a mean age of 20–50 years in both sexes (1). Patients possessing low systemic immunity, such as those with diabetes, malignant tumors, and long-term diarrhea, are more susceptible to perianal abscesses (2). Perianal abscesses usually manifest as severe anal pain, swelling with localized erythema, and fluctuance. Timely incision and drainage are the most effective therapy. Antibiotics are used selectively in patients with an anorectal abscess complicated by cellulitis, systemic illness, or underlying immunosuppression.

Studies have demonstrated that the most common gut microbiota found in perianal abscesses are *Escherichia coli*, *Proteus vulgaris*, *Staphylococcus aureus*, *Streptococcus* species, *Bacteroides*, and *Peptostreptococcus* species (3). The microbiota is essential for physiological processes, such as the biosynthesis of vitamins and amino acids, decomposition of food components, resistance against pathogenic microorganisms, development and training of the immune system, storage of fats, and modification of nervous and immune activity (4). Interestingly, there are limited studies related to the microbiota in perianal abscesses, with the existing reports primarily using culture-based diagnostic methods or 16S PCR sequencing (5, 6). Using conventional techniques, the diagnostic approaches may only be able to uncover “the tip of the metagenomic iceberg,” resulting in inappropriate antibiotic therapy. Thus, there is an urgent need to uncover the full microbial spectrum present in perianal abscesses.

Metagenomic next-generation sequencing (mNGS) can directly employ patient specimens for pan-nucleic acid detection and is able to sequence all nucleic acids from the host and pathogenic specimens (7). In recent years, mNGS has allowed a more efficient and promising means for pathogen diagnosis and is well-suited for molecular diagnosis of polymicrobial infections (8). To date, there is a lack of research on the application of mNGS for the analysis of microbiological data from perianal abscesses.

In our study, we employed high-throughput mNGS to investigate the microbiota isolated from perianal abscesses to provide a more comprehensive understanding of the etiology of perianal abscesses and tailor antimicrobial therapies as indicated.

## RESULTS

### Clinical presentation

**Fig 1:**
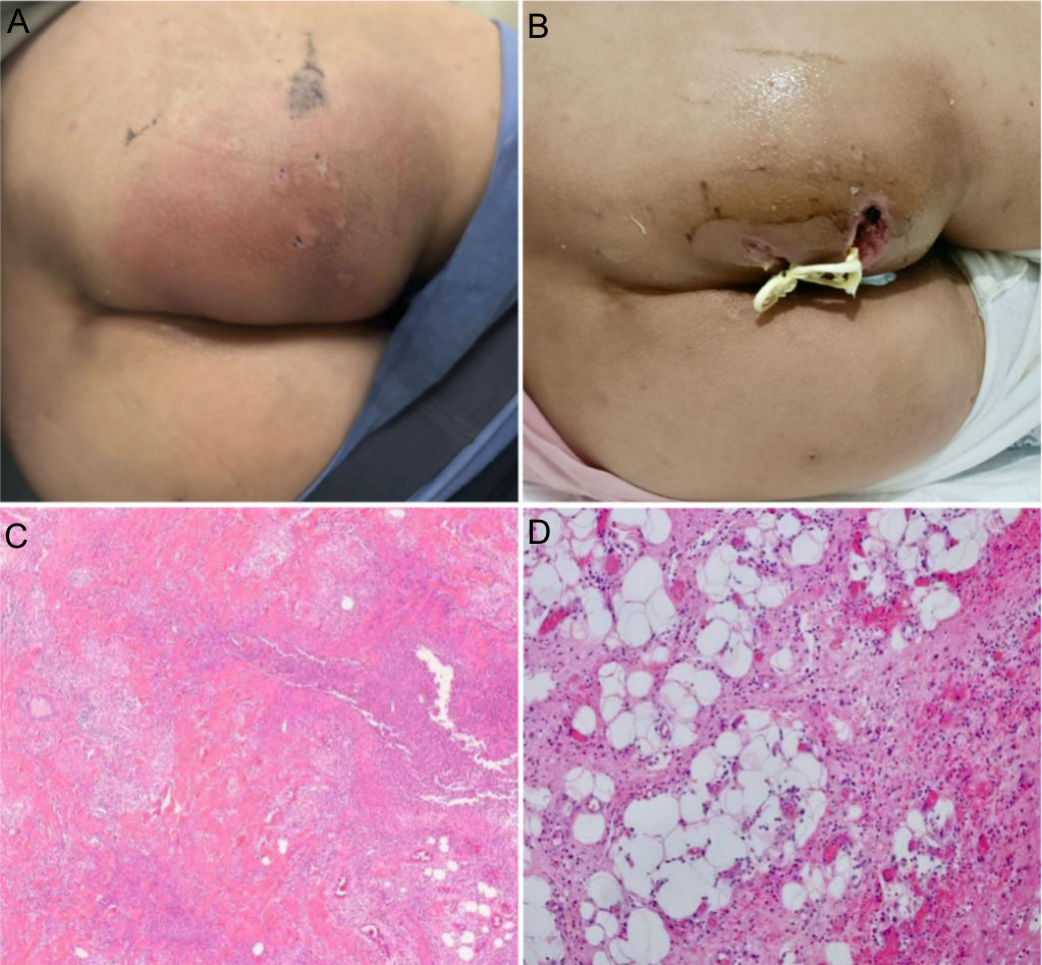
Clinical manifestations, treatment, and histological alterations in perianal abscess samples. (**A**) Typical clinical appearance of a perianal abscess (patient #7). (**B**) Incision and drainage of a perianal abscess (patient #7). (**C and D**) Numerous neutrophils and lymphocytes infiltrating surrounding dermal appendages and fibrous adipose tissue, accompanied by abscess formation in a perianal abscess sample (patient #9), were observed at 40× (**C**) and 200× (**D**) magnification, respectively.

A total of 14 fresh samples were obtained from 14 patients with perianal abscesses, aged 21–75 years, with a median age of 36.5 years. Most of the patients (10, 71.4%) were men. The clinical characteristics are outlined in Table 1. All patients presented acutely with localized, erythematous swellings with a duration of onset of 4.5 days (Fig. 1A). Some patients showed fever (5 out of 14, 35.7%). All patients possessed perianal erythematous swellings, and clinically palpable, painful masses, ranging from 4.4 cm to 21.0 cm, with an average diameter of 8.2 cm × 11.5 cm (Table 2). Three patients (21.4%) had diabetes mellitus (DM), with a mean age of 53.0 years. The DM patients were significantly older than those without DM (53.0 ± 3.0 years vs 39.6 ± 21.0 years). Additionally, two patients (14.3%) were affected by malignant tumors of the rectum. All patients received incision and drainage within 24 hours (Fig. 1B). Histologic features showed numerous neutrophils and lymphocytes infiltrating surrounding dermal appendages and fibrous adipose tissue, accompanied by abscess formation (Fig. 1C and D). Five patients (35.7%) presenting fever were treated with peri-operative broad-spectrum antibiotics. All patients were followed up over the course of 6 months and until complete recovery.

**TABLE 1:** Clinical information and culture results of perianal abscess patients^*a*^

No.	Sex	Age	Underlying diseases	Presentation	SD	WBC (10^9^/L) (3.5–9.5)	CRP (mg/L) (0–6)	Pathogen by culture	Management
1	M	21	−	Perianal swelling and pain	5	13.85	57.7	*Escherichia coli*	Incision and drainage
2	M	33	−	Perianal pain	6	12.21	28.7	*Escherichia coli*	Incision and drainage
3	F	50	Diabetes, hypertension	Fever, perianal swelling, and pain	5	10.07	160.3	−	Incision and drainage, cefoperazone sodium and sulbactam sodium
4	M	26	−	Perianal swelling and pain	3	7.17	57.3	*Escherichia coli*	Incision and drainage
5	F	67	Malignant tumors of the rectum	Fever, perianal swelling, and pain	4	28.37	297.3	*Escherichia coli*, *Bacteroides fragilis*	Incision and drainage, cefoperazone sodium and sulbactam sodium
6	M	75	Anemia	Perianal swelling and pain	4	16.44	39.4	*Streptococcus constellatus*	Incision and drainage
7	M	40	−	Recurrent perianal swelling and pain	5	8.39	16.6	*Proteus mirabilis*	Incision and drainage
8	M	72	Malignant tumors of the rectum	Fever, perianal swelling, and pain	5	11.03	121.9	*Escherichia coli*	Incision and drainage, cefoperazone sodium and sulbactam sodium
9	M	26	−	Perianal swelling and pain	3	11.54	20.5	−	Incision and drainage
10	F	26	−	Perianal swelling and pain	4	8.98	80.5	−	Incision and drainage
11	M	22	−	Perianal swelling and pain	4	9.43	27.8	*Escherichia coli*	Incision and drainage
12	M	28	−	Perianal swelling and pain	3	10.53	23.5	*−*	Incision and drainage
13	M	56	Diabetes	Fever, perianal swelling, and pain	6	12.87	95.5	*−*	Incision and drainage, cefoperazone sodium and sulbactam sodium
14	F	53	Diabetes	Fever, perianal swelling, and pain	6	12.74	112.4	−	Incision and drainage, cefoperazone sodium and sulbactam sodium

^*a*^ No., case number; SD, symptom duration before admission (days); WBC, white blood cell; CRP, C-reactive protein; M, male; F, female; and −, negative.

**TABLE 2:** Clinical characteristics for cases of perianal abscess patients

Characteristic	*N* = 14 (%)
Age, y, median (range)	36.5 (21–75)
Gender	
Male	10 (71.4)
Female	4 (28.6)
Presentation	
Fever	5 (35.7)
Erythematous swellings	14 (100.0)
Pain	14 (100.0)
Palpable mass	14 (100.0)
Size (mean)	8.2 cm × 11.5 cm
Diabetes mellitus	3 (21.4)
Malignant tumors of the rectum	2 (14.3)

Samples were acquired for culturing at the time of incision and drainage from the abscesses of 14 patients (100%). In six patients (42.9%), no growth was observed, seven patients (50.0%) had a single organism cultured from their abscesses, and only one patient (7.1%) had two microorganisms cultured (Table 1). Among the microbial cultures, *Escherichia coli* (six patients, 75.0%) was the most frequently cultured microorganism, followed by *Bacteroides fragilis*, *Streptococcus constellatus*, and *Proteus mirabilis*, each found in only one patient (12.5%) (Table 1).

### Bacterial species detected by mNGS analysis in perianal abscess samples

Table S1 outlines the bacterial species/genera alongside the read numbers for each perianal abscess sample. From the 14 samples, 2,056,684 reads were obtained with an average of 146,906 reads per sample. To avoid false positives in perianal abscess samples, we used a frequency of 1% of all reads as a threshold to cut off contaminating sequences, as reported (9). However, such a stringent threshold could potentially generate false negatives when bacterial taxa have low read numbers and relative frequency, especially from a polymicrobial sample. In our data set, this was illustrated in patient #8, who was culture-positive for *Escherichia coli*, and had 3 mNGS reads for *E. coli*, corresponding to only 0.34% of all reads. Therefore, the absolute cut-off criterion of ≥3 reads, or 0.3% of all reads, was selected to avoid false negatives.

**Fig 2:**
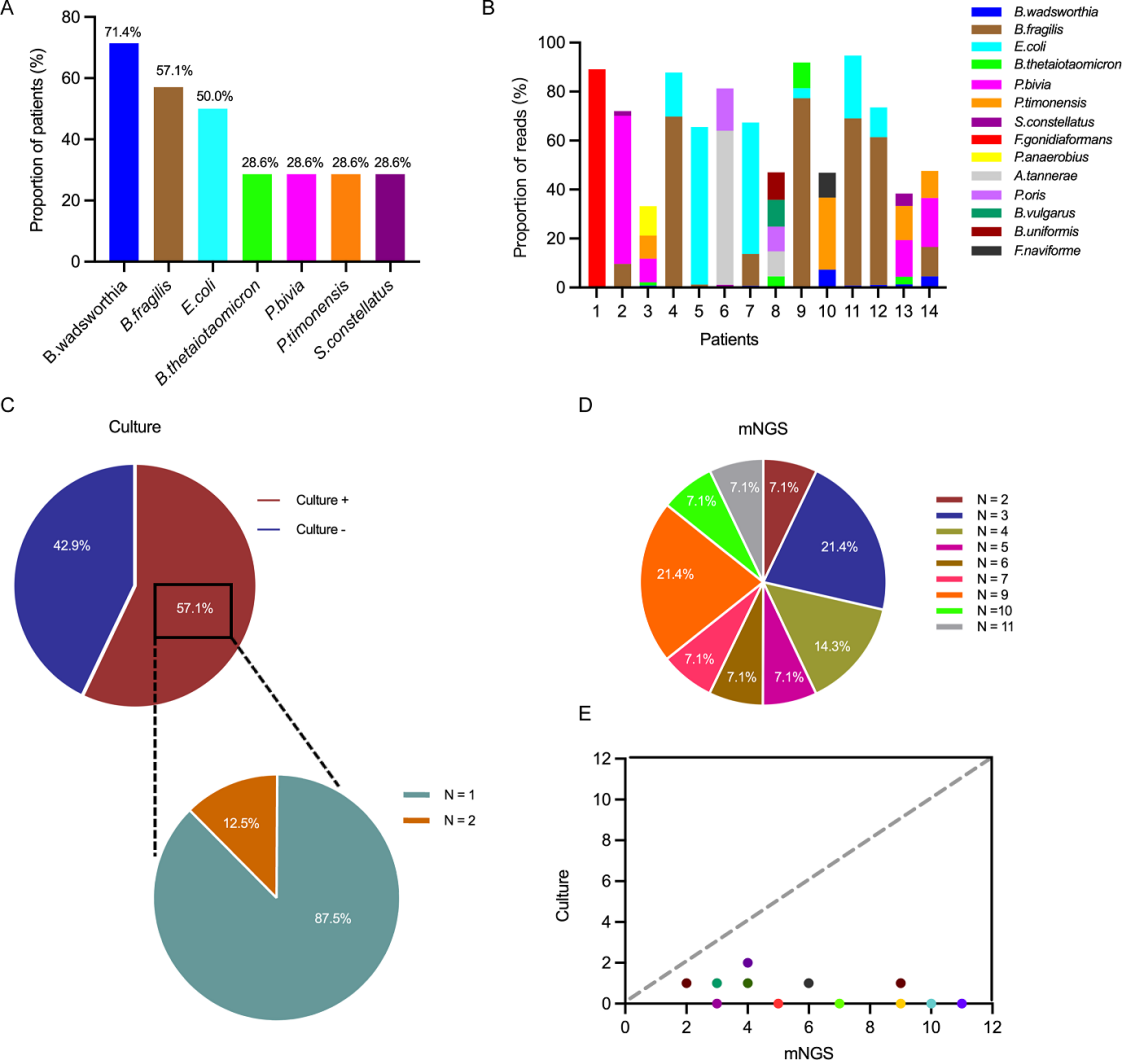
Results of metagenomic next-generation sequencing. (**A**) Distribution of pathogens in perianal abscess samples. (**B**) Pathogen information in detail from perianal abscess samples. (**C**) The proportions of bacterial growth by culture. (**D**) The proportions of bacteria identified from 2 to 11 different species were analyzed by mNGS. (**E**) Comparison of bacterial taxa number per sample identified by culturing and mNGS analysis. Each dot in (**E**) represents one perianal abscess sample. Colors were arbitrarily assigned to the samples and used to increase the visibility of individual symbols. Data points below the dashed grey line indicate increased bacterial taxa from mNGS analysis.

Using the cut-off values of 0.3% or 3 reads, a total of 40 bacterial taxa were detected at the species or genus level (Table 3). Sequencing was able to detect microorganisms in 100% of the perianal abscess samples. Gram-negative bacilli were the most predominant isolated class. The most common taxa identified by mNGS analysis were *Bilophila wadsworthia*, *Bacteroides fragilis*, and *Escherichia coli*, detected in 10 (71.4%), 8 (57.1%), and 7 (50.0%) of the 14 perianal abscess samples, respectively (Fig. 2A). The next most common bacterial species were *Bacteroides thetaiotaomicron*, *Prevotella bivia*, *Prevotella timonensis*, and *Streptococcus constellatus*, each identified in 28.6% (*n* = 4) of the perianal abscess patients (Fig. 2A).

**TABLE 3:** Overview of bacterial taxa found in perianal abscess samples

Genus	Species	Patients	Samples, *n* (%)	Genus	Species	Patients	Samples, *n* (%)
Gram-negative bacilli				Gram-positive cocci			
*Bacteroides*	*B. fragilis*	P2, P4, P6, P7, P9, P11, P12, P14	8 (57.1)	*Streptococcus*	*S. constellatus*	P2, P6, P13, P14	4 (28.6)
	*B. thetaiotaomicron*	P3, P8, P9, P13	4 (28.6)		*S. intermedius*	P2	1 (7.1)
	*B. vulgatus*	P8	1 (7.1)		*S. agalactiae*	P4	1 (7.1)
	*B. uniformis*	P8	1 (7.1)		*S. salivarius*	P5	1 (7.1)
*Prevotella*	*P. bivia*	P2, P3, P13, P14	4 (28.6)	*Finegoldia*	*F. magna*	P3, P9, P13	3 (21.4)
	*P. timonensis*	P3, P10, P13, P14	4 (28.6)	*Atopobium*	*A. minutum*	P3, P10, P13	3 (21.4)
	*P. oris*	P6, P8	2 (14.3)	*Bifidobacterium*	*B. longum*	P3, P13	2 (14.3)
	*P. stercorea*	P1	1 (7.1)		*B. breve*	P3, P13	2 (14.3)
	*P. denticola*	P6	1 (7.1)	*Slackia*	*S. exigua*	P6, P10	2 (14.3)
	*P. buccae*	P6	1 (7.1)	*Enterococcus*	*E. faecalis*	P2	1 (7.1)
*Bilophila*	*B. wadsworthia*	P3, P4, P6, P7, P9, P10, P11, P12, P13, P14	10 (71.4)		*E. avium*	P3	1 (7.1)
*Escherichia*	*E. coli*	P4, P5, P7, P8 P9, P11, P12	7 (50.0)	*Clostridium*	*C. bolteae*	P8	1 (7.1)
*Fusobacterium*	*F. nucleatum*	P6, P14	2 (14.3)		*C. symbiosum*	P8	1 (7.1)
	*F. necrophorum*	P1	1 (7.1)	*Coprococcus*	*C. eutactus*	P9	1 (7.1)
	*F. mortiferum*	P2	1 (7.1)		*C. catus*	P9	1 (7.1)
	*F. gonidiaformans*	P1	1 (7.1)	*Peptostreptococcus*	*P. anaerobius*	P3	1 (7.1)
	*F. naviforme*	P10	1 (7.1)	*Anaerococcus*	*A. obesiensis*	P3	1 (7.1)
*Alloprevotella*	*A. tannerae*	P6, P8, P14	3 (21.4)	*Flavonifractor*	*F. plautii*	P8	1 (7.1)
*Proteus*	*P. mirabilis*	P7	1 (7.1)	*Parabacteroides*	*P. distasonis*	P9	1 (7.1)
*Faecalibacterium*	*F. prausnitzii*	P9	1 (7.1)	*Peptoniphilus*	*P. harei*	P9	1 (7.1)

The seven pathogens described above were shown in each patient together alongside other organisms representing more than 10% of all reads (Fig. 2B). As highlighted in Fig. 2B, while *B. wadsworthia* (71.4%) was most detected across patients, it presented a significantly lower abundance in these patients with an average abundance of 1.7%, whereas *B. fragilis* and *E. coli* exhibited significantly higher abundance, accounting for 38.7% and 25.5%, respectively (Fig. 2B; Table S1). We also observed that *Fusobacterium gonidiaformans* (patient #1), *Prevotella bivia* (patient #2), *Prevotella timonensis* (patient #10), *Peptostreptococcus anaerobius* (patient #3), *Alloprevotella tannerae* (patient #6), and *Prevotella oris* (patient #6) were dominant in the microbial population isolated from the perianal abscess patients (Fig. 2B; Table S1).

### mNGS identifies the perianal abscess metagenome members

In culture-positive perianal abscess patients (8/14, 57.1%), conventional culturing yielded a single specific organism in 7 out of 8 (87.5%) samples and had a polymicrobial pattern in 1 out of 8 (12.5%) samples (Table 1; Fig. 2C). Employing the criteria outlined above, we observed that all perianal abscess samples were composed of at least two (and up to 11) different bacterial species as identified by mNGS with an average of 6.1 (Table 3; Fig. 2D). Thus, all perianal abscesses were polymicrobial using the mNGS methodology. All bacterial taxa identified through culturing a given sample were identified through mNGS, except in patients #1 and #2. In contrast, in all samples, mNGS identified additional bacterial taxa (Fig. 2E). Therefore, mNGS analysis of perianal abscess samples enhanced the rate of bacterial identification from perianal abscess samples.

## DISCUSSION

The cryptoglandular theory of perianal abscess formation has been accepted widely. Perianal abscesses form primarily due to infection originating in the anal crypts and migrate downward into the anal glands of the intersphincteric plane (10, 11). Limited investigations have looked into the microbiology of perianal abscesses and only using conventional culturing or 16S gene sequencing (3, 5, 6, 12, 13). This study outlines the microbiologic profile of perianal abscesses based on mNGS. To the best of our knowledge, this is the first such report originating from China.

In our study, the bacterial metagenomic landscape in perianal abscess samples investigated by mNGS contained 40 different taxa, revealing increased complexity with respect to composition compared to culturing (3, 5). The spectrum of bacteria identified ranged from well-described Gram-negative intestinal bacteria (e.g., *Escherichia coli*, *Bacteroides* spp., *Bilophila wadsworthia*) to Gram-positive bacteria (e.g., *Streptococcus* spp., *Enterococcus* spp.), but also included several species not commonly associated with perianal abscesses, (e.g., *Fusobacterium gonidiaformans* and *Alloprevotella tannerae*) (5, 6).

Several previous studies have found *E. coli* to be the predominant pathogen (14–16). Liu et al. and Eykyn et al. also reported that *E. coli* and *Bacteroides* spp. were the leading pathogens present throughout the general population (3, 17). Consistent with their research findings, our study confirmed *E. coli* and *Bacteroides fragilis* as the predominant species, followed by *Bilophila wadsworthia. Bacteroides* are involved in the metabolic regulation of the body, and *B. fragilis* is the most common, often isolated from clinical specimens, and is considered to be the most virulent *Bacteroides* species (18). Interestingly, our data indicated that although *Bilophila wadsworthia* was low in abundance, it was the most frequently (71.4%) detected species among perianal patients. Previous studies indicated that *Bilophila*, as a sulfur-producing bacterium in the intestine, was also closely linked to other intestinal diseases (19). Moreover, other studies have suggested that *Bilophila wadsworthia* increases intestinal barrier defect, bile acid dysmetabolism, changes in microbiome functional profile, and systemic inflammation (20). Therefore, as previously noted in the literature, *Bilophila wadsworthia* may be used as a potential biomarker for the diagnosis of perianal abscesses, which are closely related to inflammation (6).

*Prevotella* species, often inhabiting the human gut, are associated with gut inflammation and occupied 15% of all pathogens (6/40) identified by our study. *Prevotella* species were reported as one of the predominant anaerobes, alongside *Bacteroides fragilis*, *Peptostreptococcus* spp., *Fusobacterium* spp., *Porphyromona*s spp., and *Clostridium* spp. across the 144 perirectal abscess patients (21). Another study indicated that *Prevotella* species are involved in local and systemic infections, but the extent of their involvement and specific pathogenesis is unclear (22). Our evidence supported that *Prevotella* spp. may function in perianal abscess formation. *Alloprevotella tannerae* and *Fusobacterium gonidiaformans* were identified at high levels in patient #6 and patient #1, suggesting these two microorganisms should be focused on, and their roles in perianal abscess formation require further study. *Klebsiella pneumoniae* and *Pseudomonas aeruginosa* have been reported to cause perianal infections, but there were no instances found in this study, even among patients with diabetes mellitus (16).

Among Gram-positive bacteria, *Streptococcus* and *Enterococcus* were reported frequently in perianal abscesses, and we observed similar results in our study. Yin et al. showed that *Streptococcus* and *Enterococcus* were dominant and differed significantly after surgery for perianal abscesses, suggesting their role in the pathogenesis of perianal abscesses (6). Compared to Gram-negative bacteria, the detection rate of Gram-positive bacteria was significantly lower, suggesting that Gram-negative bacteria are the most common causative pathogens in perianal infection. All Gram-negative bacteria identified were anaerobic. The antimicrobial coverage of anaerobes could allow for better control of perianal infection.

In the context of mNGS, the potential for amplification of cross-contaminating bacterial DNA during sample processing is a notable concern, leading to challenges in distinguishing genuine signals from contamination (9). To mitigate this issue, we sought guidance from prior research, which proposed the establishment of a proportional cut-off criterion of >1% of all reads (9). However, specific bacterial taxa identified by culture in our study exhibited minimal read counts (*n* = 3), constituting approximately 0.34% of the total. Given this scenario, we opted for an absolute cut-off criterion, stipulating a minimum of ≥3 reads or 0.3%. This adjustment aimed to reduce the risk of false negatives, recognizing that it may not entirely eliminate the potential for discerning true signals from contaminants.

In all cases, mNGS identified more numerous bacterial taxa, with an average of 6.1 compared to traditional culture-based methods, which only detected an average of 1.1 in culture-positive perianal abscess patients. The types of bacteria detected through culturing were minimal, representing predominantly *E. coli* (75.0%), restricted to medium selectivity or antibiotics (23). Similar to brain, lung, and liver abscesses, caused by multiple kinds of bacteria, perianal abscesses are more complex than previously thought (24–26). In this study, 10 out of 14 samples had two or more microorganisms with an abundance over 10%, with most being opportunistic, suggesting pathogen heterogeneity and bacterial co-infection in perianal abscesses. Meanwhile, mNGS technology enabled the detection of drug resistance genes in *E. coli* (in patients #4, #5, and #9), *B. fragilis* (in patient #4), and *S. constellatus* (in patient #6), playing a crucial role in guiding the selection of antibiotic treatment strategies and predicting treatment outcomes. Moreover, compared to CSF culture, mNGS dramatically reduced the diagnostic duration to less than 36 hours. However, in 2 out of 14 samples, bacteria detected by culturing were not found in the mNGS analysis. This could be due to reduced efficiency lysis of the cell wall of bacteria during the processing of mNGS samples (9).

Our study has several limitations. The sample size included was relatively small, with only three diabetic patients needing to be individually analyzed because the microorganisms of perianal abscesses in diabetes patients may differ from those in immunocompetent patients (3). Upon mNGS analysis, because of the very short sequencing length, an unequivocal identification at the species level may not be possible when highly similar or related species are identified. Moreover, because of its retrospective design, selection bias may exist for obtaining culture samples.

In conclusion, mNGS-based metagenomic analysis of perianal abscesses revealed a more diverse bacterial profile involving bacterial co-infections compared to microbiological culture and suggested the bacteria *Bilophila wadsworthia* could be a potential biomarker for perianal abscesses. However, more detailed aspects of these findings should be clarified in future studies to further characterize the differential gut microbiota and verify the effectiveness of *Bilophila wadsworthia* as a biomarker.

## MATERIALS AND METHODS

### Participants

Based on the diagnostic criteria, patients were diagnosed with perianal abscesses by two gastroenterologists and registered for this study from March 2023 to August 2023 (1). These patients were admitted to the anorectal surgery department of the First Affiliated Hospital of Fujian Medical University. Inclusion criteria for the patients included the diagnosis of the first episode of a perianal abscess and those aged 18 years or older. Exclusion criteria for the patients included concomitant anorectal fistula, secondary or recurrent perianal abscess, previous perianal surgery, inflammatory bowel disease, history of radiation to the pelvic area, severe cardiovascular diseases, pregnancy, and local skin trauma or infections.

All bacterial cultures obtained from perianal abscesses were cultured under both aerobic and anaerobic conditions in the Laboratory Medicine Center at the First Affiliated Hospital of Fujian Medical University. Pathogens were identified based on colonial morphology as well as standard biochemical tests.

Data obtained from the medical records included patients’ demographics, including age, gender, co-morbidities, clinical characteristics, and duration of hospital stay, as well as laboratory data, including white blood cell count and inflammatory markers, pus culture results, and antibiotic treatments.

### Metagenomic next-generation sequencing

#### Sample collection

Pus obtained from all participants were collected in 10-mL sterile centrifuge tubes at the time of incision and drainage. The samples were frozen within 4 hours of extraction and stored at −80°C. Repeated freezing and thawing were not permitted, and the entire process relied on transport using dry ice. All frozen samples were processed within 6 months.

#### Nucleic acid extraction

Samples were sealed aseptically and transported on dry ice to Hugobiotech Co., Ltd. (Beijing, China) for mNGS detection. DNA was extracted and purified by removing a sample of 200 µL of cell-free supernatant based on the instructions of the QIAamp DNA Micro Kit (Qiagen, Hilden, Germany). DNA concentration and quality were measured using a Qubit 3.0 Fluorometer (Invitrogen, Q33216) and agarose gel electrophoresis (Major Science, UVC1-1100).

#### Library generation and sequencing

DNA library construction was conducted using the Qiagen Library Construction Kit (QIAseq Ultralow Input Library Kit) operating instructions. Library quality control was assessed using a Qubit 3.0 Fluorometer (Invitrogen, Q33216) and Agilent 2100 Bioanalyzer (Agilent Technologies, Palo Alto, USA). DNA libraries possessing different barcode tags were pooled and sequenced using the Illumina Nextseq 550 sequencing platform (Illumina, San Diego, USA) with an SE75bp sequencing strategy.

#### Bioinformatics pipeline

After acquiring sequencing data, high-quality data were obtained by filtering out adaptors, low quality, low complexity, and short sequences. Human-derived sequences matching the human reference database (hg38) were disregarded using SNAP software. The remaining data were aligned to the microbial genome database using Burrow-Wheeler Alignment. This database contained more than 30,000 microbial genomes from NCBI, with 17,748 species of bacteria, 11,058 viruses, 1,134 species of fungi, and 308 species of parasites. The microbial composition of the samples was determined. The criteria for the mNGS result were established as follows: (i) for bacteria outside of TB, fungi other than Cryptococcus, and parasites, the following conditions were applied: either sequencing coverage ranked within the top 10 of all detected and undetected pathogens when compared to the negative control (NTC), or a sample/NTC ratio with a reads per million (RPM) value exceeding 10. (ii) For viruses, tuberculosis, and Cryptococcus, one of the following criteria was met: the identification of at least one specific sequence distinct from the NTC or a sample/NTC RPM ratio surpassing 5.

## Data Availability

All sequence data filtering out the human genome of this study have been uploaded to the National Center for Biotechnology Information sequence read archives under project accession number PRJNA1067536.

## SUPPLEMENTAL MATERIAL

Table S1 spectrum.03474-23-s0001.docx Detailed information of bacterial taxa identified in mNGS.

ASM does not own the copyrights to Supplemental Material that may be linked to, or accessed through, an article. The authors have granted ASM a non-exclusive, world-wide license to publish the Supplemental Material files. Please contact the corresponding author directly for reuse.
