# Supplementary material for: Microbiota in adult perianal abscess revealed by metagenomic next-generation sequencing
Source: Microbiol Spectr. 2024 Feb 22;12(4):e03474-23. doi: 10.1128/spectrum.03474-23 (PMC10986498; doi:10.1128/spectrum.03474-23)
Supplement: Table S1 — Detailed information of bacterial taxa identified in mNGS. [file spectrum.03474-23-s0001.docx]

**Supplementary Data**

**Microbiota in adult perianal abscess revealed by metagenomic next-generation sequencing**

**Jian-Chen Hong^1,2#^,** **Jian-Sheng Chen^1#^, Zai-Jie Jiang^3^,** **Zhi-Chuan Chen^1^, Ning Ruan^1,2^*, Xiang-Ping Yao^3,4^****

^1^Department of Gastrointestinal Surgery, the First Affiliated Hospital, Fujian Medical University, Fuzhou 350005, China

^2^Department of Anorectal Surgery, National Regional Medical Center, Binhai Campus of the First Affiliated Hospital, Fujian Medical University, Fuzhou 350212, China

^3^Department of Neurology and Institute of Neurology of First Affiliated Hospital, Institute of Neuroscience, and Fujian Key Laboratory of Molecular Neurology, Fujian Medical University, Fuzhou 350005, China

^4^Department of Neurology, National Regional Medical Center, Binhai Campus of the First Affiliated Hospital, Fujian Medical University, Fuzhou 350212, China

**^#^** These authors contributed equally

*Corresponding Author. Department of Gastrointestinal Surgery, the First Affiliated Hospital, Fujian Medical University, China. E-mail address: 476006754@qq.com

******Corresponding Author. Department of Neurology, The First Affiliated Hospital, Institute of Neuroscience, Fujian Key Laboratory of Molecular Neurology, Fujian Medical University, Fuzhou 350005, China. E-mail address: yaoxiangping@fjmu.edu.cn

**Table S1** Detailed information of bacterial taxa identified in mNGS.

| Patient | Sex | Age | Culture | mNGS Results | Reads | % |
| --- | --- | --- | --- | --- | --- | --- |
| 1 | M | 21 | *Escherichia coli* | *Prevotella stercorea* | 5 | 0.64 |
|  |  |  |  | *Fusobacterium necrophorum* | 24 | 4.50 |
|  |  |  |  | *Fusobacterium gonidiaformans* | 378 | 89.12 |
| 2 | M | 33 | *Escherichia coli* | *Bacteroides fragilis* | 122 | 9.65 |
|  |  |  |  | *Prevotella bivia* | 371 | 60.51 |
|  |  |  |  | *Fusobacterium mortiferum* | 9 | 1.39 |
|  |  |  |  | *Streptococcus constellatus* | 9 | 1.88 |
|  |  |  |  | *Streptococcus intermedius* | 39 | 8.42 |
|  |  |  |  | *Enterococcus faecalis* | 3 | 0.36 |
| 3 | F | 50 | - | *Bacteroides thetaiotaomicron* | 90 | 1.41 |
|  |  |  |  | *Prevotella bivia* | 246 | 9.63 |
|  |  |  |  | *Prevotella timonensis* | 306 | 9.39 |
|  |  |  |  | *Bilophila wadsworthia* | 35 | 0.75 |
|  |  |  |  | *Finegoldia magna* | 45 | 2.33 |
|  |  |  |  | *Atopobium minutum* | 10 | 0.57 |
|  |  |  |  | *Bifidobacterium longum* | 162 | 6.88 |
|  |  |  |  | *Bifidobacterium breve* | 106 | 4.61 |
|  |  |  |  | *Enterococcus avium* | 25 | 0.55 |
|  |  |  |  | *Peptostreptococcus anaerobius* | 257 | 12.04 |
|  |  |  |  | *Anaerococcus obesiensis* | 24 | 1.16 |
| 4 | M | 26 | *Escherichia coli* | *Bacteroides fragilis* | 4382 | 69.27 |
|  |  |  |  | *Bilophila wadsworthia* | 27 | 0.48 |
|  |  |  |  | *Escherichia coli* | 1010 | 18.06 |
|  |  |  |  | *Streptococcus agalactiae* | 9 | 0.35 |
| 5 | F | 67 | *Escherichia coli* | *Escherichia coli* | 347 | 64.37 |
|  |  |  | *Bacteroides fragilis* | *Streptococcus salivarius* | 3 | 1.17 |
| 6 | M | 75 | *Streptococcus constellatus* | *Bacteroides fragilis* | 143 | 0.01 |
|  |  |  |  | *Prevotella oris* | 134873 | 17.31 |
|  |  |  |  | *Prevotella denticola* | 60351 | 8.12 |
|  |  |  |  | *Prevotella buccae* | 13886 | 1.67 |
|  |  |  |  | *Bilophila wadsworthia* | 207 | 0.02 |
|  |  |  |  | *Fusobacterium nucleatum* | 8949 | 1.55 |
|  |  |  |  | *Alloprevotella tannerae* | 411200 | 62.83 |
|  |  |  |  | *Streptococcus constellatus* | 5420 | 1.09 |
|  |  |  |  | *Slackia exigua* | 3358 | 0.63 |
| 7 | M | 40 | *Proteus mirabilis* | *Bacteroides fragilis* | 145 | 12.96 |
|  |  |  |  | *Bilophila wadsworthia* | 7 | 0.72 |
|  |  |  |  | *Escherichia coli* | 523 | 53.70 |
|  |  |  |  | *Proteus mirabilis* | 12 | 1.51 |
| 8 | M | 72 | *Escherichia coli* | *Bacteroides thetaiotaomicron* | 17 | 4.23 |
|  |  |  |  | *Bacteroides vulgarus* | 36 | 10.91 |
|  |  |  |  | *Bacteroides uniformis* | 34 | 11.28 |
|  |  |  |  | *Prevotella oris* | 20 | 10.17 |
|  |  |  |  | *Escherichia coli* | 3 | 0.34 |
|  |  |  |  | *Alloprevotella tannerae* | 16 | 10.01 |
|  |  |  |  | *Clostridium bolteae* | 12 | 2.95 |
|  |  |  |  | *Clostridium symbiosum* | 3 | 0.88 |
|  |  |  |  | *Flavonifractor plautii* | 17 | 6.97 |
| 9 | M | 26 | - | *Bacteroides fragilis* | 1172774 | 77.18 |
|  |  |  |  | *Bacteroides thetaiotaomicron* | 158259 | 10.51 |
|  |  |  |  | *Bilophila wadsworthia* | 20 | 0.10 |
|  |  |  |  | *Escherichia coli* | 60222 | 4.10 |
|  |  |  |  | *Faecalibacterium prausnitzii* | 481 | 0.10 |
|  |  |  |  | *Finegoldia magna* | 80 | 0.10 |
|  |  |  |  | *Coprococcus eutactus* | 751 | 0.08 |
|  |  |  |  | *Coprococcus catus* | 123 | 0.01 |
|  |  |  |  | *Parabacteroides distasonis* | 1791 | 0.10 |
|  |  |  |  | *Peptoniphilus harei* | 235 | 0.10 |
| 10 | F | 26 | - | *Prevotella timonensis* | 114 | 29.35 |
|  |  |  |  | *Bilophila wadsworthia* | 41 | 7.33 |
|  |  |  |  | *Fusobacterium naviforme* | 28 | 10.21 |
|  |  |  |  | *Atopobium minutum* | 7 | 3.37 |
|  |  |  |  | *Slackia exigua* | 6 | 2.37 |
| 11 | M | 22 | *Escherichia coli* | *Bacteroides fragilis* | 3561 | 68.27 |
|  |  |  |  | *Bilophila wadsworthia* | 27 | 0.80 |
|  |  |  |  | *Escherichia coli* | 1201 | 25.61 |
| 12 | M | 28 | *-* | *Bacteroides fragilis* | 4834 | 60.34 |
|  |  |  |  | *Bilophila wadsworthia* | 54 | 1.00 |
|  |  |  |  | *Escherichia coli* | 890 | 12.22 |
| 13 | M | 56 | *-* | *Bacteroides thetaiotaomicron* | 90 | 3.07 |
|  |  |  |  | *Prevotella bivia* | 498 | 15.03 |
|  |  |  |  | *Prevotella timonensis* | 470 | 13.87 |
|  |  |  |  | *Bilophila wadsworthia* | 35 | 1.30 |
|  |  |  |  | *Streptococcus constellatus* | 142 | 5.07 |
|  |  |  |  | *Finegoldia magna* | 45 | 1.42 |
|  |  |  |  | *Atopobium minutum* | 10 | 0.31 |
|  |  |  |  | *Bifidobacterium longum* | 162 | 5.87 |
|  |  |  |  | *Bifidobacterium breve* | 106 | 4.23 |
| 14 | F | 53 | - | *Bacteroides fragilis* | 533 | 12.0 |
|  |  |  |  | *Prevotella bivia* | 974 | 20.05 |
|  |  |  |  | *Prevotella timonensis* | 461 | 11.09 |
|  |  |  |  | *Bilophila wadsworthia* | 207 | 4.51 |
|  |  |  |  | *Fusobacterium nucleatum* | 89 | 2.90 |
|  |  |  |  | *Alloprevotella tannerae* | 33 | 0.81 |
|  |  |  |  | *Streptococcus constellatus* | 54 | 1.46 |

Abbreviation: M, male; F, female; -, negative.
